# High yield purification of *Helicobacter pylori* neutrophil-activating protein overexpressed in *Escherichia coli*

**DOI:** 10.1186/s12896-015-0136-x

**Published:** 2015-04-08

**Authors:** Yu-Chi Yang, Ting-Yu Kuo, Zhi-Wei Hong, Han-Wen Chang, Chung-Chu Chen, Te-Lung Tsai, Hua-Wen Fu

**Affiliations:** Institute of Molecular and Cellular Biology, National Tsing Hua University, Hsinchu, 30013 Taiwan, Republic of China; Department of Internal Medicine, Division of Hepatology and Gastroenterology, Mackay Memorial Hospital, Hsinchu, 30055 Taiwan, Republic of China; Department of Pathology and Laboratory Medicine, Mackay Memorial Hospital, Hsinchu, 30055 Taiwan, Republic of China; Department of Life Science, National Tsing Hua University, Hsinchu, 30013 Taiwan, Republic of China

**Keywords:** HP-NAP, *Helicobacter pylori*, *E. coli*, DEAE, Sephadex, Negative chromatography, Batch chromatography, pH, Solubility, ROS

## Abstract

**Background:**

*Helicobacter pylori* neutrophil-activating protein (HP-NAP) is involved in *H. pylori*-induced gastric inflammation. Due to its immunogenic and immunomodulatory properties, HP-NAP has been used for developing vaccines against *H. pylori* infection and new drugs for cancer therapy.

**Results:**

Here, we provide a simple process for high-yield production of HP-NAP by applying one-step negative chromatography to purify recombinant HP-NAP expressed in *Escherichia coli* (*E. coli*). In our *E. coli* expression system, recombinant HP-NAP constitutes nearly 70% of the total protein. Overexpressed recombinant HP-NAP is almost completely soluble upon cell lysis at pH 9.5. Under the optimal condition at pH 8.0, recombinant HP-NAP with purity higher than 95% can be obtained from *E. coli* by collecting the unbound fraction using diethylaminoethyl (DEAE) Sephadex resin in batch mode. The overall yield of HP-NAP from a 50-ml *E. coli* culture is ~19 mg. The purified HP-NAP folds into a multimer with a secondary structure of α-helix and is able to trigger the production of reactive oxygen species by neutrophils.

**Conclusions:**

Purification of recombinant HP-NAP overexpressed in *E. coli* using DEAE Sephadex negative mode batch chromatography is an efficient method for high-yield production of highly pure HP-NAP in its native state. The purified HP-NAP is useful for various clinical applications including vaccine development, diagnosis, and new drug development.

## Background

*Helicobacter pylori* (*H. pylori*) colonizes the gastric mucosa of human stomach for over half of the entire population. The majority of the infected people are asymptomatic with moderate inflammation. However, the infected people have a 10 to 20% of lifetime risk of developing peptic ulcer, and ~1% of risk of acquiring gastric cancer [1,2]. *H. pylori*-induced chronic active inflammation is characterized by the infiltration of various inflammatory cells, such as polymorphonuclear leukocytes, lymphocytes, monocytes, and macrophages, at the gastric mucosa [3,4]. *Helicobacter pylori* neutrophil-activating protein (HP-NAP), a major virulence factor of *H. pylori*, could play a key role in recruitment and activation of these immune cells during *H. pylori* infection.

HP-NAP was first identified by its ability to promote the production of reactive oxygen species (ROS) by neutrophils and the adhesion of neutrophil to endothelial cells [5]. This protein is a spherical dodecamer composed of identical subunits [6]. Each subunit is a four-α-helix bundle protein with a molecular weight of approximately 17 kDa [6,7]. HP-NAP released from the bacterium may cross the stomach epithelial cells and endothelial cells in the stomach [8-10]. Upon encountering the neutrophils, HP-NAP directly activates and recruits them to the site of infection [9,11]. In addition to the induction of ROS production, HP-NAP stimulates neutrophils to secrete several chemokines, including CXCL8/interleukin-8 (IL-8), CCL3/macrophage inflammatory protein 1 alpha (MIP-1α), and CCL4/MIP-1β [10], which can recruit and activate additional neutrophils, monocytes, and lymphocytes. HP-NAP also induces the secretion of tumor necrosis factor alpha (TNF-α), IL-6, and IL-8 by monocytes [12,13]. Furthermore, HP-NAP is a potent immunomodulator to induce a polarized T helper type 1 (Th1) immune response and to trigger the release of proinflammatory cytokines, including IL-12, TNF-α, interferon gamma (IFN-γ) [13]. These immune responses induced by HP-NAP may contribute to the pathological outcome of *H. pylori* infection.

Despite its pathogenic role, HP-NAP can be used to develop vaccines against *H. pylori* and novel therapeutic agents to treat cancer, as well as allergic and infectious diseases [11,14]. With regard to vaccine development, immunization with the multi-protein vaccine containing HP-NAP, cytotoxin-associated gene A (CagA), and vacuolating cytotoxin A (VacA) in beagle dogs has a therapeutic effect to induce humoral immune response and reduce the colonization of bacterium in gastric mucosa [15]. This protein vaccine has been evaluated for its safety and immunogenicity in a clinical trial [16]. HP-NAP has also been delivered by attenuated viral vectors as a recombinant protective antigen in mice [17]. These findings support the idea that HP-NAP can be used to develop vaccines for *H. pylori* immunoprophylaxis in humans. In the development of novel therapeutic agents, recombinant HP-NAP encoded by attenuated measles viral vectors has a therapeutic effect on metastatic breast cancer in mice [18]. Recombinant HP-NAP expressed from oncolytic adenovirus has also been shown to have an antitumor ability against neuroendocrine tumors *in vivo* [19]. Furthermore, in a mouse model of bladder cancer implant, peritumoral injection of HP-NAP reduced the burden and vascularization of the tumor [20]. In addition to cancer therapy, administration of HP-NAP to mice inhibits the Th2 responses in ovalbumin-induced allergic asthma and *Trichinella spiralis* infection [21,22]. Therefore, HP-NAP is a potent immunomodulator to trigger Th1-polarized immune responses for cancer therapy and to down regulate Th2-mediated immune responses elicited by allergic reactions and parasitic infections. Thus, HP-NAP could be used in clinical therapy.

Several methods for purification of recombinant HP-NAP expressed in *Escherichia coli* (*E. coli*) in its native state have been reported. In these reported methods, at least two chromatographic steps, including gel-filtration chromatography, are required to obtain pure HP-NAP [23-25]. However, recombinant HP-NAP expressed in *Bacillus subtilis* (*B. subtilis*) has been reported to be purified by using one-step diethylaminoethyl (DEAE) anion-exchange chromatography through the collection of the unbound fraction at pH 8.0 [26]. Due to the low expression level of HP-NAP in *B. subtili*s, the overall yield of recombinant HP-NAP was not satisfactory. In our *E. coli* expression system, HP-NAP was highly overexpressed [25]. Since the endogenous proteins in different expression systems are different, we re-investigate whether the same approach can be applied to purify recombinant HP-NAP expressed in *E. coli* in one step. By optimization of the conditions for cell lysis and purification, we herein report a simple method using DEAE Sephadex negative mode batch chromatography for high yield purification of recombinant HP-NAP expressed in *E. coli*.

## Results

### Effect of pH on solubility of HP-NAP in *E. coli* lysate

**Figure 1:**
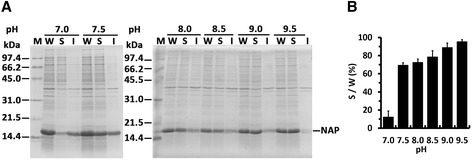
**The effect of pH on the solubility of recombinant HP-NAP upon** ***E. coli*** **lysis. A**, *E. coli* BL21(DE3) expressing HP-NAP was suspended in ice-cold Tris–HCl buffer at the indicated pH ranging from 7.0 to 9.5. Cells were then disrupted by sonication as described in Methods. Whole cell lysates (W) were centrifuged to separate soluble fractions (S) and insoluble pellets (I). The proteins were analyzed by SDS-PAGE. Molecular weights (M) in kDa are indicated at the side of the gels. **B**, The percentage of solubility of recombinant HP-NAP in the whole cell lysate at each pH was calculated from the intensity of HP-NAP band on SDS gels for the soluble fraction (S) divided by that for the whole cell lysate (W). Data were represented as the mean ± S.D. of at least two experiments.

To improve the solubility of HP-NAP during the cell lysis stage, *E. coli* expressing recombinant HP-NAP was lysed in Tris buffer with pH ranging from 7.0 to 9.5. As shown in Figure 1, overexpressed recombinant HP-NAP was detected as a major band with an apparent molecular weight of 17 kDa by SDS-PAGE. At pH 7.0, little amount of recombinant HP-NAP was present in the soluble fraction (Figure 1). The amount of HP-NAP detected in the soluble fraction was markedly increased at pH 7.5 to 9.5 (Figure 1). At pH 9.5, HP-NAP was mainly present in the soluble fraction (Figure 1). This result indicates that HP-NAP is highly soluble in *E. coli* lysate at pH 9.5, which is far from the reported pI, 6.75, of HP-NAP [7].

### Optimization of the negative purification of HP-NAP expressed in *E. coli*

**Figure 2:**
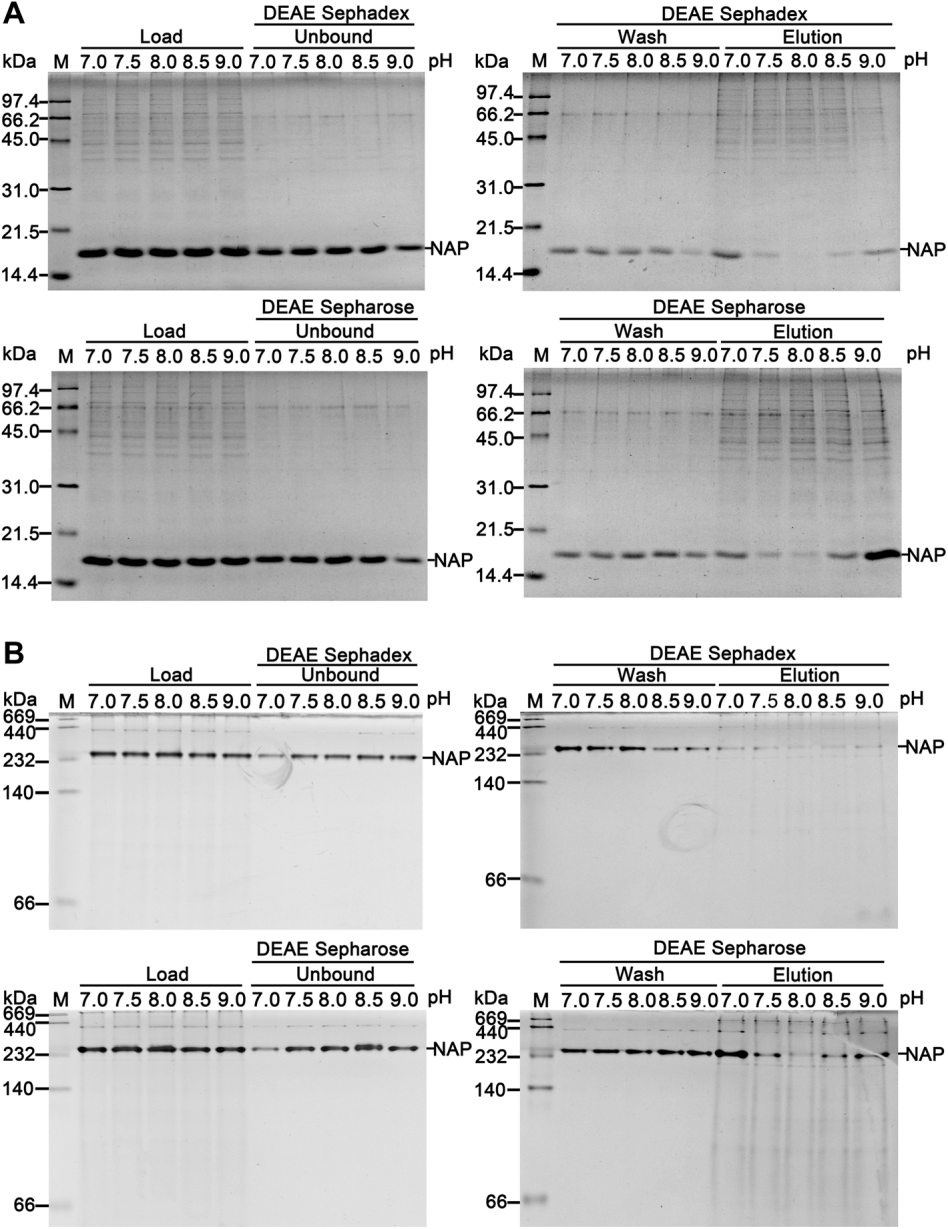
**Purification of recombinant HP-NAP expressed in** ***E. coli*** **by DEAE resins at pH 7.0 to 9.0.** Soluble fractions of *E. coli* expressing HP-NAP lysed at pH 9.0 were adjusted to the indicated pH ranging from 7.0 to 9.0 and a protein concentration of 0.3 mg/ml. These adjusted fractions, indicated as load, were then loaded onto DEAE Sephadex and DEAE Sepharose resins to purify recombinant HP-NAP by a batch method at 4°C as described in Methods. The unbound, wash, and elution fractions were analyzed by SDS-PAGE **(A)** and native-PAGE **(B)**. Molecular weights (M) in kDa are indicated on the left of the stained gels. Similar results were obtained in at least two to four independent experiments.

Our previous study has shown that recombinant HP-NAP expressed in *B. subtilis* can be obtained in high purity by negative purification through the collection of the unbound fraction using DEAE Sephadex anion-exchange resin at pH 7.5 and 8.0 [26]. Since the upper limit of the pH working range for DEAE is 9.0, we here re-investigated whether the same approach can be used to isolate highly pure recombinant HP‐NAP expressed in *E. coli* over a wider pH range of 7.0 to 9.0. The soluble fraction containing recombinant HP-NAP isolated from *E. coli* lysate at pH 9.0 and those adjusted to pH 8.5, 8.0, 7.5, and 7.0 were subjected to purification by DEAE Sephadex and DEAE Sepharose anion-exchange resins. At pH 7.5 to 8.5, the majority of the recombinant HP-NAP remained in the unbound fraction for both resins (Figure 2A). At pH 8.0, only little amount of recombinant HP-NAP was detected in the elution fraction (Figure 2A). Also, the amount of recombinant HP-NAP in the elution fraction for DEAE Sepharose resin was higher than that for DEAE Sephadex resin (Figure 2A), indicating that DEAE Sepharose resin is capable of binding more recombinant HP-NAP than DEAE Sephadex resin. However, at pH 7.0 and 9.0, recombinant HP-NAP was mainly present in the elution fraction for both resins (Figure 2A). At all pH values investigated, recombinant HP-NAP was expressed as an oligomeric protein with an apparent molecular weight of ~232 kDa by native-PAGE analysis (Figure 2B). Due to the stronger binding ability of DEAE Sepharose resin to the recombinant HP-NAP at pH 8.0, the overall yield of the recombinant HP-NAP obtained from the unbound fraction using DEAE Sepharose resin could be less. Thus, DEAE Sephadex resin was chosen to purify recombinant HP-NAP expressed in *E. coli* through the collection of the unbound fraction at pH 8.0.

**Figure 3:**
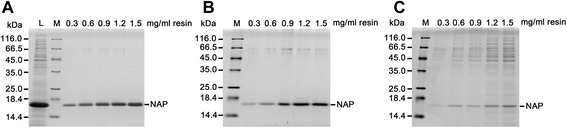
**Optimization of the amount of proteins loaded onto resins for purifying recombinant HP-NAP from** ***E. coli*** **.** The soluble proteins from the whole cell lysate of *E. coli* BL21(DE3) expressing HP-NAP were loaded onto DEAE Sephadex resins according to the indicated ratio of mg proteins per milliliter of resins to purify recombinant HP-NAP by a batch method at pH 8.0 as described in Methods. The soluble protein, indicated as load (L), the unbound fraction **(A)**, wash fraction **(B)**, and elution fraction **(C)** were analyzed by SDS-PAGE. Molecular weights (M) in kDa are indicated on the left of the stained gels. Similar results were obtained in two independent experiments.

We next determined an optimal loading ratio of the amount of soluble proteins from *E. coli* lysate loaded onto DEAE Sephadex resin at pH 8.0 to obtain a maximum yield of highly pure recombinant HP-NAP by collecting the unbound fraction. At the ratios of 0.3 to 1.5 mg of proteins per milliliter of DEAE Sephadex resins being tested, the purified recombinant HP-NAP was present in the unbound and wash fractions (Figure 3A and B). Most of the impure proteins from *E. coli* were present in the elution fractions. The amount of impure proteins in the elution fraction was increased as the ratio of protein to resin increased (Figure 3C). Even though the amount of recombinant HP-NAP was increased in the unbound fraction as the ratio of protein to resin increased (Figure 3A), the amount of recombinant HP-NAP and impure proteins in the elution fraction was not markedly increased when the ratio of protein to resin reached 1.5 mg/ml (Figure 3C). This result suggests that the binding capacity of DEAE Sephadex resin has reached its maximum level at the ratio of 1.5 mg of proteins per milliliter resins. Thus, the optimized amount of proteins loaded onto DEAE Sephadex resin should be 1.5 mg of proteins per milliliter resins for purification of recombinant HP-NAP expressed in *E. coli*.

### Purification of HP-NAP expressed in *E. coli* by negative mode batch chromatography using DEAE Sephadex anion-exchange resin

**Figure 4:**
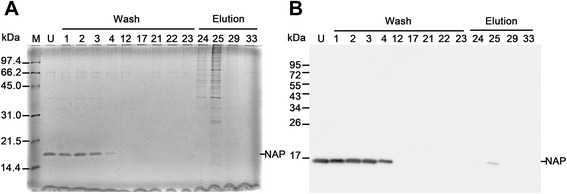
**Purification of recombinant HP-NAP from** ***E. coli*** **by DEAE Sephadex negative mode batch chromatography.** The soluble fraction from the whole cell lysate of *E. coli* BL21(DE3) expressing HP-NAP was subjected to DEAE Sephadex anion-exchange chromatography as described in Methods. The fractions of wash and elution ranged from 1 to 23 and 24 to 33, respectively. The unbound fraction (U), selected wash fractions, and selected elution fractions were analyzed by SDS-PAGE **(A)** and immunoblotting **(B)**. Molecular weights (M) in kDa are indicated on the left of the stained gel and the blot. Data were representative of at least two independent experiments.

**Figure 5:**
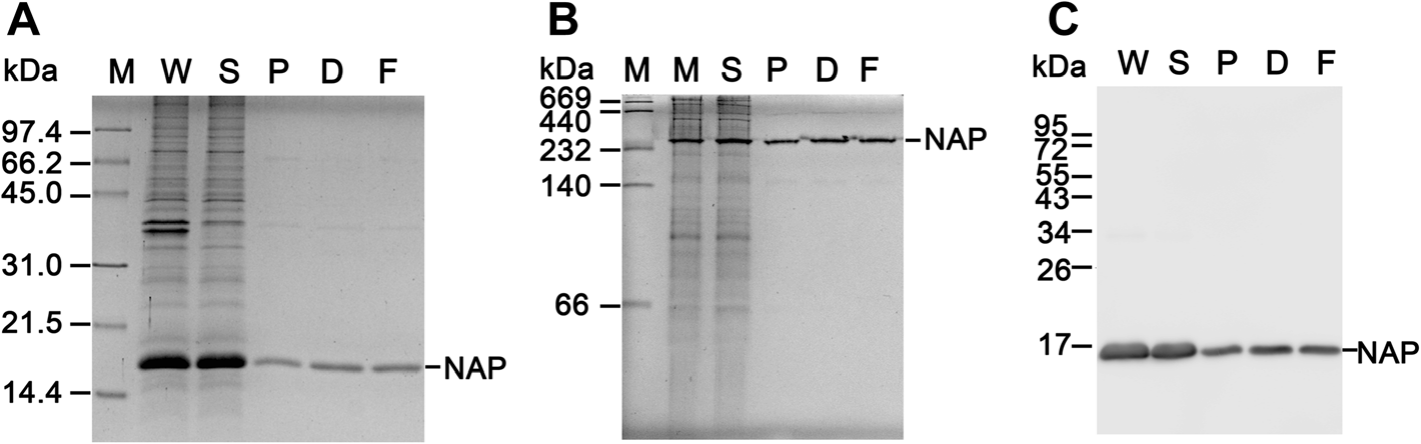
**PAGE and immunoblotting analysis of the purification process of recombinant HP-NAP expressed in** ***E. coli*** **.** The whole cell lysate (W) and soluble fraction (S) of *E. coli* BL21(DE3) expressing HP-NAP, the pooled fraction (P) of the unbound fraction and wash fractions containing HP-NAP from DEAE Sephadex chromatography, the dialyzed HP-NAP solution (D), and the filtrated HP-NAP solution (F) were analyzed by SDS-PAGE **(A)**, native-PAGE **(B)**, and immunoblotting **(C).** Molecular weights (M) in kDa are indicated on the left of the stained gels and the blot. Data were representative of at least three independent experiments.

Recombinant HP-NAP overexpressed in *E. coli* was subjected to DEAE Sephadex negative mode batch chromatography at pH 8.0 with the ratio of protein to resin being 1.5 mg/ml. As shown in Figure 4A, the majority of recombinant HP-NAP remained in the unbound fraction and some residual recombinant HP-NAP was present in the wash fractions. Only little amount of recombinant HP-NAP was present in the elution fraction as detected by immunoblotting (Figure 4B). The unbound fraction and the wash fractions containing recombinant HP-NAP were pooled. After the pooled fraction was subjected to dialysis for buffer exchange to Dulbecco’s phosphate-buffered saline (D-PBS), pH 7.2, and to syringe filtration for endotoxin removal, the purity of recombinant HP‐NAP was further increased (Figure 5A and Table 1) and the amount of endotoxin was less than 2.22 endotoxin unit (EU)/mg protein. The purified recombinant HP-NAP was an oligomeric protein with an apparent molecular weight of ~232 kDa by native-PAGE analysis (Figure 5B) and was specifically recognized by the monoclonal antibody against HP-NAP by immunoblot analysis (Figure 5C). A typical result for purification of HP-NAP expressed in *E. coli* is shown in Table 1. The purity of HP-NAP was 95.97% after the step of DEAE Sephadex chromatography, by which a recovery of 90.5% was achieved. After dialysis and syringe filtration, the final yield was 132.4 mg HP-NAP per gram of cell paste from *E. coli* culture, and the final purity of HP-NAP achieved was 99.32% with a total recovery of 82.1%. This result reveals that negative purification of recombinant HP-NAP expressed in *E. coli* using DEAE Sephadex anion-exchange resin at pH 8.0 is able to obtain recombinant HP-NAP in one step with high purity and high yield.

**Table 1:** **Purification summary table of recombinant HP-NAP expressed in** ***E. coli*** ^**a**^

**Purification step**	**Total protein (mg)** ^**b**^	**Volume (ml)**	**Purity (%)** ^**c**^	**Amount of HP-NAP (mg)** ^**d**^	**Step recovery of HP-NAP (%)** ^**e**^	**Total recovery of HP-NAP (%)** ^**f**^
Whole cell lysate	26.70	20.00	69.45	18.54	100	100
Supernatant	28.60	20.00	73.34	20.98	113	113
DEAE Sephadex chromatography	19.78	59.95	95.97	18.98	90.5	102
Dialysis	18.54	57.95	97.12	18.01	94.9	97.1
Acrodisc syringe filtration	15.33	56.79	99.32	15.23	84.6	82.1

^a^From 0.115 g of *E.coli* cell paste obtained from 50 ml culture.

^b^Protein concentration determined by the Bradford method with bovine serum albumin (BSA) as the reference.

^c^Values determined from densitometry measurement as described in Methods.

^d^Values determined by multiplying the values in the column of “Total protein” and “Purity”.

^e^Values determined by dividing the amount of HP-NAP from each purification step by that from the previous step.

^f^Values determined by dividing the amount of HP-NAP from each purification step by that from the whole cell lysate.

### Structural and functional characterization of purified HP-NAP

**Figure 6:**
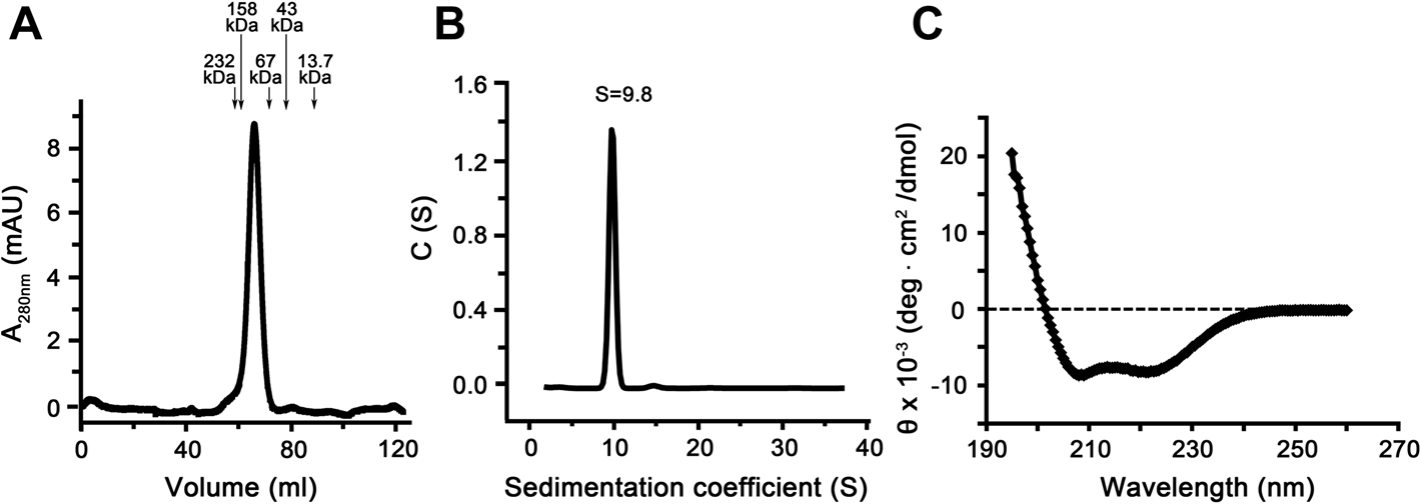
**Molecular and structural properties of purified recombinant HP-NAP expressed in** ***E. coli*** **.** The apparent molecular weight, sedimentation coefficient, and secondary structure of HP-NAP in D-PBS, pH 7.2, were analyzed by gel filtration, analytical ultracentrifugation, and CD spectroscopy, respectively, as described in Methods. **A**, The UV absorbance was recorded at 280 nm for the elution from a HiLoad 16/60 Superdex 200 prep grade gel gel-filtration column. The molecular weights of the protein markers were indicated at the chromatogram. **B**, The sedimentation coefficient distribution c(s) was shown as a function of S. The c(s) distribution was analyzed using the software program SEDFIT. **C**, The far UV CD spectrum of HP-NAP was recorded at the wavelength range of 195 to 260 nm. Data were representative of at least three independent experiments.

The structural and molecular properties of the purified recombinant HP-NAP were examined by gel filtration, analytical ultracentrifugation, and circular dichroism (CD) spectroscopy to confirm its proper folding. By gel filtration analysis, recombinant HP-NAP was eluted as a single peak with the molecular weight of about 150 kDa (Figure 6A), which is consistent with the previous report [5] but is much lower than the molecular weight of ~232 kDa as determined by native-PAGE analysis (Figure 2B and 5B) and the theoretical molecular weight of 203 kDa of dodecameric HP-NAP. The low apparent molecular weight of HP-NAP determined by gel filtration may be due to a more compact overall shape of HP-NAP as compared with those of the standard proteins used for calibration. The high apparent molecular weight of HP-NAP determined by native-PAGE may be resulted from a less negative net charge of HP-NAP. By analytical ultracentrifugation analysis, recombinant HP-NAP was sedimented as a single peak with a sedimentation coefficient of 9.8 S (Figure 6B). These findings indicate that recombinant HP-NAP purified from *E. coli* is assembled into an oligomeric protein. Structure analysis from CD spectroscopy showed that the recombinant HP-NAP was mainly composed of α-helices (Figure 6C). Thus, the purified recombinant HP-NAP obtained by negative purification using DEAE Sephadex anion-exchange resin retained its oligomeric status with α-helical structure.

**Figure 7:**
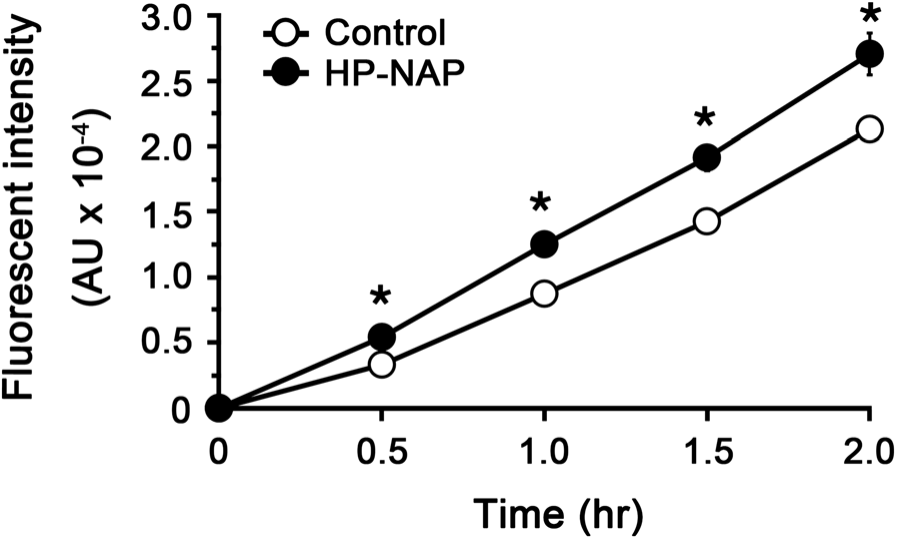
**Production of ROS by neutrophils stimulated with recombinant HP-NAP determined by H**_**2**_ **DCFDA-derived fluorescence assay.** Human neutrophils (1 x 10^5^ cells) were treated with 0.5 μM HP-NAP and D-PBS, pH 7.2, as a negative control at 37°C. The content of ROS generated from neutrophils was measured continuously by using H_2_DCFDA-derived fluorescence assay as described in Methods. Data were represented as the mean ± S.D. of one experiment in triplicate (*, *p*<0.05). Similar results were obtained in two independent experiments.

**Figure 8:**
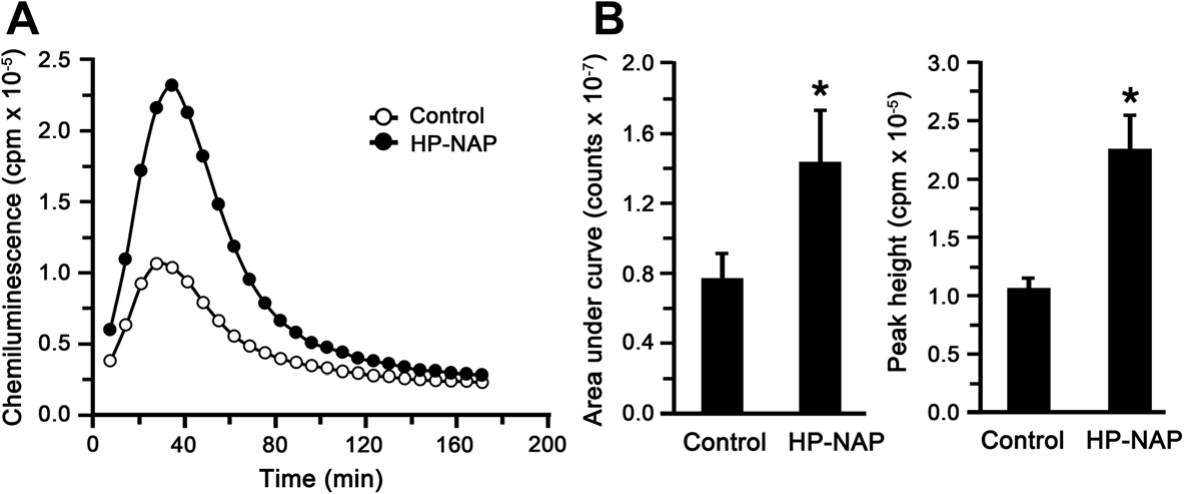
**Production of ROS by neutrophils stimulated with recombinant HP-NAP determined by luminol-dependent chemiluminescence assay. A**, Human neutrophils (1 x 10^5^ cells) were treated with 0.5 μM HP-NAP and D-PBS, pH 7.2, as a negative control at 37°C. The content of ROS generated from neutrophils was measured continuously by using luminol-dependent chemiluminescence assay as described in Methods. **B**, The integral chemiluminescence signals calculated from the area under curve and the peak value of chemiluminescence responses of neutrophils stimulated with HP-NAP were used to compare with the control. Data were represented as the mean ± S.D. of one experiment in triplicate (*, *p*<0.05). Similar results were obtained in two independent experiments.

The ability of recombinant HP-NAP purified from *E. coli* to induce ROS production by human neutrophils was examined by 2’,7’-dihydrodichlorofluorescein diacetate (H_2_DCFDA)-derived fluorescence and luminol-dependent chemiluminescence assays. H_2_DCFDA-derived fluorochrome was used as an indicator of intracellular ROS level generated by neutrophils. The production of ROS was significantly increased by at least 1.3 fold in cells stimulated with HP-NAP as compared to the control cells (Figure 7). Luminol-dependent chemiluminescence was used to measure the generation of both extracellular and intracellular ROS by neutrophils upon HP-NAP stimulation. The pattern of HP-NAP-induced luminol-dependent chemiluminescence is shown in Figure 8A. The stimulation of recombinant HP-NAP caused a significant burst respiration of neutrophils in comparison with the control as indicated by the increased chemiluminescence signals of both the area under the curve and the peak value (Figure 8B and C). Therefore, recombinant HP-NAP purified from *E. coli* is capable of activating human neutrophils to produce ROS.

## Discussion

In this study, the effect of pH on negative purification of recombinant HP-NAP overexpressed in *E. coli* using DEAE anion-exchange resins was investigated. At pH 7.0, most of the recombinant HP-NAP bound to DEAE resins, while at pH ranging from 7.5 to 9.0, most of the recombinant HP-NAP remained in the unbound fraction. At pH 8.0, the amount of recombinant HP-NAP present in the unbound fraction reached a maximum level. Under the optimized condition at pH 8.0, recombinant HP-NAP can be obtained in one step with high purity and high yield by collecting the unbound fraction from batch chromatography using DEAE Sephadex anion-exchange resin. The purified recombinant HP-NAP kept its α-helical oligomeric structure and was capable of stimulating neutrophils to produce ROS.

Recombinant HP-NAP expressed in *E. coli* purified by anion-exchange chromatography has been reported to be present in the flow-through fraction. DEAE Sepharose resin and Q Sepharose resin were used to purify recombinant HP-NAP expressed in *E. coli* by collecting the flow-through fraction at pH 7.5 and pH 8.0, respectively [23,24]. Thus, the ionizable ligands of the resin and the pH of the buffer may serve as two important parameters to ensure that HP-NAP is present in the flow-through rather than binds to the resin. However, both studies show that an extra gel filtration chromatographic step is required to achieve higher purity. Our finding that less HP-NAP bound to DEAE Sephadex resin than DEAE Sepharose resin indicates that the matrix of the resin also affects the protein binding. In this study, we first optimized the pH of the buffer to allow the maximum amount of HP-NAP was present in the unbound fraction. Then, an appropriate amount of the soluble proteins from *E. coli* lysates loaded onto DEAE Sephadex resin at pH 8.0 was further adjusted to ensure the maximum binding of the impure proteins from *E. coli* to the resin so that one-step negative purification of highly pure recombinant HP-NAP from *E. coli* could be achieved.

Our results show that purification of recombinant HP-NAP with more than 95% purity and 90% recovery was achieved at the step of DEAE Sephadex chromatography (Table 1). The yield of HP-NAP from a 50-ml *E. coli* culture was 18.9 mg. Since the purity of recombinant HP-NAP in the *E. coli* lysate was ~70% (Table 1), such a high level expression of recombinant HP-NAP in *E. coli* should increase the concentration and purity of HP-NAP in the flow-through fraction, resulting in a high recovery and high purity of recombinant HP-NAP using the purification method presented here. In our other reports, even though recombinant HP-NAP was expressed in the same system, the purity of HP-NAP obtained by using the two-step gel-filtration chromatography was 98% [25], and the yield of HP-NAP was 91.5 mg per liter of *E. coli* culture [27]. There is at least a 4-fold increase in the yield of recombinant HP-NAP purified from *E. coli* using this negative chromatography with DEAE Sephadex anion-exchange resin as compared to the two-step gel filtration chromatography. The batch chromatography using DEAE Sephadex anion-exchange resin also reduces the purification time as compared to the gel-filtration chromatography. Therefore, DEAE Sephadex negative mode batch chromatography presented in this study is a simple and efficient method to purify recombinant HP-NAP expressed in *E. coli*.

## Conclusions

In this study, a method of negative chromatography using DEAE Sephadex anion-exchange resin in batch mode has been established to purify recombinant HP-NAP expressed in *E. coli* in one step. After optimization of the conditions of cell lysis and purification, highly pure recombinant HP-NAP in its native state can be obtained in high yield. HP-NAP purified from this approach should be beneficial for the development of vaccines, drugs, or diagnostics against *H. pylori*.

## Methods

### Expression of recombinant HP-NAP in *E. coli*

*E. coli* BL21(DE3) harboring an expression plasmid of pET42a-NAP encoding a *nap* gene from *H. pylori* strain 26695 [GenBank:AE000543.1, Gene: HP0243] prepared as previously described [25] was used for protein expression. The bacteria were streaked on a LB agar plate containing 50 μg/ml kanamycin, and then the plate was incubated at 37°C for 16 hr. A single colony was picked and inoculated into 5 ml of LB broth containing 50 μg/ml kanamycin. The preculture was incubated at 37°C for 16 hr with shaking at 170 rpm. A volume of 3 ml of the above preculture was then inoculated into the 300 ml of LB broth containing 50 μg/ml kanamycin, and the culture was incubated at 37°C for 2 hr with shaking at 170 rpm until the absorbance at 600 nm reached approximately 0.4-0.5. Isopropyl β-D-1-thiogalactopyranoside (IPTG) was added into the culture to a final concentration of 0.4 mM for induction of the expression of HP-NAP. The culture was incubated for 3 hr until the absorbance at 600 nm reached approximately 1.6 to 1.7. Afterwards, the cells were centrifuged at 6,000 × g at 4°C for 15 min to remove the supernatant, and the pellet was preserved at −70°C until purification.

The above *E. coli* cell pellets from 9 ml of culture were resuspended in one-third of the culture volume of 20 mM Tris buffer (Tris–HCl, pH 7.0, 7.5, 8.0, 8.5, 9.0, and 9.5), 50 mM NaCl, and 0.1% (v/v) protease inhibitor mixture (PI mix) at 4°C. The PI mix contained 0.13 M phenylmethylsulfonyl fluoride (PMSF), 0.3 M N-alpha-tosyl-L-lysinyl-chloromethylketone (TLCK), and 0.3 M N-tosyl-L-phenylalaninyl-chloromethylketone (TPCK). The bacterial suspensions were disrupted by an ultrasonic processor SONICS VCX-750 (Sonics & Materials, Newtown, CT, USA) on ice at 20% amplitude with 5 sec on and 10 sec off pulses for 4.5 min. The lysate was centrifuged at 30,000 × g at 4°C for 1 hr to separate insoluble and soluble proteins by using a himac CS150NX table-top microcentrifuge with a S80AT3 rotor (Hitachi Koki Co. Ltd., Tokyo, Japan). The proteins in whole cell lysates and soluble and insoluble fractions were examined by SDS-PAGE using gels containing 15% acrylamide.

### Adjustment of pH of the soluble fraction of *E. coli* lysate at 4°C

The pH value of the soluble fraction from *E. coli* lysate in 20 mM Tris–HCl, pH 9.0, and 50 mM NaCl with the addition of PI mix was kept at pH 9.0 or adjusted to pH 8.5, 8.0, 7.5, and 7.0 with the addition of HCl. The volume of HCl added to the fraction was according to the proportion of 40 ml of buffer at pH 9.0 adjusted to pH 8.5, 8.0, 7.5, pH 7.0 at 4°C and was less than 1% of the fraction volume. In brief, to achieve the desired pH, 1 ml of soluble fraction at pH 9.0 was added with the following: 3.63 ul of 1 N HCl for pH 8.5, 8.88 μl of 1 N HCl for pH 8.0, 6.44 μl of 2 N HCl for pH 7.5, and 7.31 μl of 2 N HCl for pH 7.0. For keeping the same protein concentration in each fraction, these soluble fractions were then added with 20 mM Tris–HCl and 50 mM NaCl at their respective pH values to make the total volume added to each fraction to be 8.88 μl. Protein concentrations were routinely analyzed by the Bradford method using a commercial dye preparation (Bio‐Rad, Hercules, CA, USA), and BSA was used as a standard.

### Optimization of the purification of recombinant HP-NAP by DEAE anion-exchange resins

For the experiment to optimize the pH, the soluble fractions from *E. coli* lysate with a protein concentration of 0.3 mg/ml at pH 9.0 and at the adjusted pH values of 8.5, 8.0, 7.5, and 7.0 were loaded onto DEAE Sephadex A-25 (Sigma-Aldrich, St. Louis, MO, USA) and DEAE Sepharose (Amersham Pharmacia Biotech, Uppsala, Sweden) resins, which were pre-equilibrated with 20 mM Tris–HCl and 50 mM NaCl at the same pH value of the soluble fractions. For the experiment to optimize the amount of proteins loaded onto the resins, the soluble fraction in 20 mM Tris–HCl, pH 8.0, and 50 mM NaCl with a protein concentration of 0.1, 0.2, 0.3, 0.4, and 0.5 mg/ml were loaded onto DEAE Sephadex A-25 and DEAE Sepharose resins, which were pre-equilibrated with 20 mM Tris–HCl, pH 8.0, and 50 mM NaCl. The volume ratio of the soluble fraction to the resin was 3:1. The protein/resin slurries were shaken on a rotator for 30 min at 4°C to ensure the binding of proteins to the resins. The slurries were centrifuged at 10,000 × *g* at 4°C for 30 sec, and the supernatant was retained as the unbound fraction. An equal resin volume of 20 mM Tris–HCl and 50 mM NaCl at the same pH value of the soluble fractions was added to both DEAE resins, and the slurries were shaken on a rotator for 10 min to wash out the proteins nonspecifically binding to the resins. The slurries were centrifuged at 10,000 × *g* at 4°C for 30 sec, and the supernatant was retained as the wash fraction. After washing five times, an equal resin volume of 20 mM Tris–HCl and 1 M NaCl at the same pH value of the soluble fraction was added to both DEAE resins, and then the slurries were shaken on a rotator for 10 min to elute the proteins binding to the resins. The slurries were centrifuged at 10,000 × *g* at 4°C for 30 sec, and the supernatant was retained as the elution fraction. This elution step was repeated three times. The fractions including the unbound fraction, wash fraction, and elution fraction were analyzed by SDS-PAGE and native-PAGE using gels containing 15% and 10% acrylamide, respectively.

### Purification of recombinant HP-NAP expressed in *E. coli* by negative mode batch chromatography using DEAE Sephadex anion-exchange resin

The cell pellet harvested from 50 ml of the culture of *E. coli* expressing recombinant HP-NAP was resuspended in 20 ml of ice-cold 20 mM Tris–HCl, pH 8.0, and 50 mM NaCl with the addition of PI mix. The bacterial suspension was disrupted by a EmulsiFlex-C3 high pressure homogenizer (Avestin Inc., Ottawa, Canada) at 17,000 psi for 7 passes. The cell lysate was centrifuged at 30,000 × *g* at 4°C for 1 hr to separate soluble proteins and insoluble debris. The soluble protein fraction with a protein concentration of 0.5 mg/ml was incubated with 7 ml of DEAE Sephadex A-25 resin, and protein/resin slurries were mixed by shaking on a rotator at 4°C for 1 hr. The volume ratio of the soluble protein fraction to the resin was 3:1. The slurries were then placed into a column, and the column was run by gravity flow at 4°C. The solution that flowed through the column was collected as the unbound fraction. The column was washed with five resin volumes of 20 mM Tris–HCl, pH 8.0, and 50 mM NaCl. The volume of each collected wash fraction was 1 ml for the first resin volume and 1.75 ml for the rest. The proteins binding to the resins were eluted by the addition of three resin volumes of 20 mM Tris–HCl, pH 8.0, and 1 M NaCl, and the elutes were collected in 2-ml fractions. The fractions of unbound, wash, and elution were analyzed by SDS-PAGE and immunoblotting.

For obtaining a maximum amount of the recombinant HP-NAP, the wash fractions with protein concentration of at least 0.1 mg/ml were pooled together with the unbound fraction. The pooled fraction containing purified recombinant HP-NAP was dialyzed against D-PBS, pH 7.2, with 100-fold volume of the pooled fraction for 4 hr at 4°C by using Spectrum/Por dialysis tubing with molecular weight cutoff of 14 kDa (Spectrum Laboratories, Rancho Dominguez, CA, USA) and dialyzed again overnight with the fresh buffer. After dialysis, the purified recombinant HP-NAP was subjected to endotoxin removal by filtering through a syringe with a positively-charged, hydrophilic Mustang E membrane at a flow rate ranging from 2.5 to 4 ml/min. The amount of endotoxin present in recombinant HP-NAP was less than 2.22 EU/mg protein as determined by Super Laboratory Company (Taipei, Taiwan) using an enzyme-linked immunosorbent assay (ELISA) with the detection limit of 0.005 to 2 EU/ml. In addition to SDS-PAGE, purified recombinant HP-NAP was routinely analyzed by gel filtration chromatography and native-PAGE as described previously [26] to confirm its oligomeric properties. The percentage purity of HP-NAP was calculated from the intensities of protein bands on SDS gels as follows: purity (%) = (intensity of HP-NAP)/(intensity of total proteins) × 100. The intensities of protein bands were quantified by densitometry analysis using multi gauge software V3.0 (Fujifilm, Tokyo, Japan).

### Immunoblotting

The protein samples were separated by a 15% SDS-PAGE gel and then transferred onto a polyvinylidene difluoride (PVDF) membrane. The membrane was incubated in 5% nonfat milk in Tris-buffered saline/Tween-20 (TBST) containing 50 mM Tris‐HCl, pH 7.4, 15 mM NaCl, and 0.1% Tween‐20 at room temperature for 1 hr to block nonspecific binding. The membrane was then probed with hybridoma culture supernatants containing mouse monoclonal antibody MAb 16F4 against HP-NAP [17] with a dilution factor of 1:200 in TBST containing 5% BSA at 4°C overnight. After three washes with TBST containing 5% nonfat milk for 10 min each, the membrane was probed with horseradish peroxidase-conjugated mouse secondary antibody (Jackson ImmunoResearch, West Grove, PA, USA) at a dilution of 1:5000 in TBST containing 5% nonfat milk at room temperature for 1 hr and then washed with TBST three times. The signals on the immunoblot were detected by the enhanced chemiluminescence assay (ECL) Western blotting detection reagents (PerkinElmer, Waltham, MA, USA) and LAS-3000 imaging system (Fujifilm, Tokyo, Japan).

### Analytical ultracentrifugation

The sedimentation coefficient of HP-NAP in D-PBS, pH 7.2, was determined by analytical ultracentrifugation with a PorteomeLab™ XL-I protein characterization system (Beckman Coulter, Brea, CA, USA) at 116,444 × *g* (38,000 rpm) at 20°C for 240 min as previously described [26]. Sedimentation coefficient distribution, c(s), of HP-NAP was analyzed with the software program SEDFIT.

### Circular dichroism spectroscopy

The secondary structure of recombinant HP-NAP in D-PBS, pH 7.2, was measured by CD spectroscopy in the far UV region. The CD spectrum was recorded on an Aviv model 62A DS CD spectrophotometer (Aviv Biomedical, Lakewood, NJ, USA) at 25°C with a 1 mm path‐length quartz cuvette. A concentration of 0.3 mg/ml of HP-NAP was repetitively scanned from 260 to 195 nm in 1 nm increments. A 1 nm bandwidth and 1 sec averaging time were utilized. The blank-subtracted CD signals from a total of five scans were averaged. The mean residue ellipticity (MRE) was calculated from the formula: MRE= θ/(10 × L × C × N), where θ is the CD signal (mdeg), L is the path length of the cell (cm), C is the protein concentration of the sample (M), and N is the number of peptide bonds.

### Isolation of human neutrophils

Human peripheral blood was collected in a tube with sodium heparin by venipuncture from three healthy adult volunteers, who signed informed consents under the approval of the Institutional Review Board (IRB) of National Tsing-Hua University, Hsinchu, Taiwan. Neutrophils were isolated from heparinized blood by dextran sedimentation of erythrocytes, followed by Ficoll-Paque PLUS density gradient (GE Healthcare, Buckinghamshire, UK) as described previously [25] except that the final cell pellet containing neutrophils was resuspended in D-PBS, pH 7.2, containing 5 mM glucose (D-PBS-G). The cells were kept on ice and used within 5 hr. The viability of neutrophils was assessed by trypan blue dye exclusion count using a hemocytometer. The purity of neutrophils was determined by examination of at least 700 cells on each Liu’s stained cytocentrifuged slide using Zeiss Axiovert 200 microscope with 400× magnification (Carl Zeiss, Jena, Germany). The viability and purity of neutrophils in each preparation exceeded 96.1% and 95.3%, respectively.

### Measurement of reactive oxygen species

The production of ROS from human neutrophils measured by H_2_DCFDA-derived fluorescence microplate assay was essentially the same as previously described [26]. Briefly, aliquots of 50 μl of isolated human neutrophils at 2 × 10^6^ cells/ml in D‐PBS‐G were dispensed into individual wells of a 96‐well black plate (Nunc, Rochester, NY, USA) with a flat bottom. Subsequently, 150 μl of the mixture containing 0.9 mM CaCl_2_, 0.5 mM MgCl_2_, 0.27 mM H_2_DCFDA (Invitrogen, Carlsbad, CA, USA), and 0.67 μM HP-NAP in the D‐PBS, pH 7.2, was added into each well to a final volume of 200 μl. The cells were then incubated at 37°C. H_2_DCFDA was dissolved in methanol at a concentration of 10 mM as the stock solution and diluted into D-PBS, pH 7.2, containing 0.9 mM CaCl_2_ and 0.5 mM MgCl_2_ immediately before use. The final concentrations of H_2_DCFDA and recombinant HP-NAP were 0.2 mM and 0.5 μM, respectively. The emission of fluorescence at 538 nm was monitored at 37°C with excitation at 485 nm in triplicate every 30 min for 2 hr using a Wallac 1420–012 VICTOR 3 multilabel counter (Perkin-Elmer, Waltham, MA, USA). A rise in the intracellular ROS level was evaluated by the increase in the DCF fluorescence intensity calculated from the formula: ΔF= F_t_ – F_0_, where F_t_ and F_0_ represent the fluorescence intensity at time t and time 0 after incubation, respectively.

The production of ROS from human neutrophils measured by luminol-dependent chemiluminescence assay was as previously described [25] except for the addition of calcium and magnesium in the assay buffer. Briefly, aliquots of 50 μl of isolated human neutrophils at 2 × 10^6^ cells/ml in D-PBS-G were dispensed into individual wells of a 96-well white plate (Nunc, Rochester, NY, USA) with a flat bottom. Subsequently, 150 μl of the mixture containing 0.9 mM CaCl_2_, 0.5 mM MgCl_2_, 13.3 μM luminol (sigma), and 0.67 μM HP-NAP in the D‐PBS, pH 7.2, was added into each well to a final volume of 200 μl. The cells were then incubated at 37°C. Luminol was dissolved in dimethyl sulfoxide (DMSO) at a concentration of 10 mM as the stock solution and diluted into D-PBS, pH 7.2, containing 0.9 mM CaCl_2_ and 0.5 mM MgCl_2_ immediately before use. The final concentrations of luminol and recombinant HP-NAP were 10 μM and 0.5 μM, respectively. The emission of chemiluminescence was monitored in triplicate for 5 sec per well over a 3-hr period by a Wallac 1420 (Victor2) Multilabel Counter (Perkin-Elmer, Waltham, MA, USA).

### Statistical analysis

Results were expressed as mean ± standard deviation (S.D.). Statistical analysis was performed by using Excel 2010 software (Microsoft). Statistical difference was determined by two-tailed unpaired Student’s *t*-test. A probability (*p*) value which was less than 0.05 was considered statistically significant.

## Abbreviations

*B. subtilis*: *Bacillus subtilis*
BSA: Bovine serum albumin
CD: Circular dichroism
DEAE: Diethylaminoethyl
H_2_DCFDA: 2’,7’-dihydrodichlorofluorescein diacetate
D-PBS: Dulbecco’s phosphate-buffered saline
EU: Endotoxin unit
*E. coli*: *Escherichia coli*
*H. pylori*: *Helicobacter pylori*
HP-NAP: *Helicobacter pylori* neutrophil-activating protein
IL: Interleukin
MIP-1: Macrophage inflammatory protein 1
PI mix: Protease inhibitor mixture
ROS: Reactive oxygen species
SDS-PAGE: Sodium dodecyl sulfate polyacrylamide gel electrophoresis
Th: T helper
TBST: Tris-buffered saline/Tween-20
TNF-α: Tumor necrosis factor α
